# A study on the value of ultrasound strain elastography-based radiomics nomogram in the differential diagnosis of breast masses

**DOI:** 10.1038/s41598-025-29430-3

**Published:** 2025-11-27

**Authors:** Yunpei Zhu, Yanping Dou, Qifan Hu, Hui Wang

**Affiliations:** Ultrasound department of the First Affiliated Hospital of Dalian Medical University, No. 5 Longbin Road, Jinzhou District, Dalian City, Liaoning Province China

**Keywords:** Radiomics, Ultrasound, Breast masses, Strain elastography, Machine learning, Breast cancer

## Abstract

The aim of this study was to construct a radiomics nomogram for prediction of breast masses (BMs) by analyzing the clinical characteristics of the patients as well as radiomics features of two-dimensional (2D) ultrasound images and strain elastography images. In this retrospective study, 219 patients diagnosed with BMs were enrolled and randomly divided into training set and testing set in a 7:3 ratio. Radiomics nomogram was constructed based on clinical features and Radscore to compare area under the receiver operating characteristic curve (AUC) with another models. The AUCs of the training set were 0.83, 0.91, 0.92, 0.96, and 0.99 for the clinical model, elastography radiomics model, 2D radiomics model, bimodal radiomics model, and nomogram, respectively, and the AUCs of the testing set were 0.86, 0.87, 0.91, 0.93, and 0.95, respectively. There were significant differences in AUC between nomogram and another models (*p* < 0.05). 2D ultrasound radiomics model and strain elastography radiomics model were of diagnostic value in identifying BMs. The bimodal radiomics model was superior to these two single-modal radiomics models. Nomogram can further enhance the diagnosis of BMs and contribute valuable information for clinical decision making.

**Supplementary Information:**

The online version contains supplementary material available at 10.1038/s41598-025-29430-3.

## Introduction

Breast cancer (BC) accounts for 30% of female cancers, and its incidence rate is growing at about 0.5% per year^1^. Previous studies have shown that the tumour stage at the time of diagnosis of BC is a key factor affecting the prognosis of BC patients, and the prognosis varies greatly depending on the tumour stage^2^. Therefore, early detection, diagnosis and treatment of BC are crucial for improving the prognosis of patients. The methods currently used to diagnose breast masses (BMs) are mainly puncture biopsy and pathological analysis after surgical excision. Puncture biopsy is an invasive procedure and may have some sampling error. Therefore, the development of a non-invasive method to accurately and safely identify BMs remains a key challenge.

BC screening can be done in a variety of methods, such as MRI, mammography and ultrasound (US)^3^. Mammography’s diagnostic efficacy decreases in dense breast tissue, while MRI is costly and difficult to use for routine screening^4–6^. US has become the primary method for early screening of BMs due to its low cost, no radiation and real-time availability.

Strain elastography is a semi-quantitative elastography US imaging method that estimates tissue stiffness based on tissue displacement from either external (manual pressurisation of the transducer) or patient sources (physiological movements of the heart and respiration), and then converts the gradient of displacement into pixels for imaging^7^. Currently sonographers mainly use strain ratios and elastography scores to estimate tissue stiffness, which has been shown to have good sensitivity and specificity for detecting MBMs^8,9^.

**Fig. 1:**
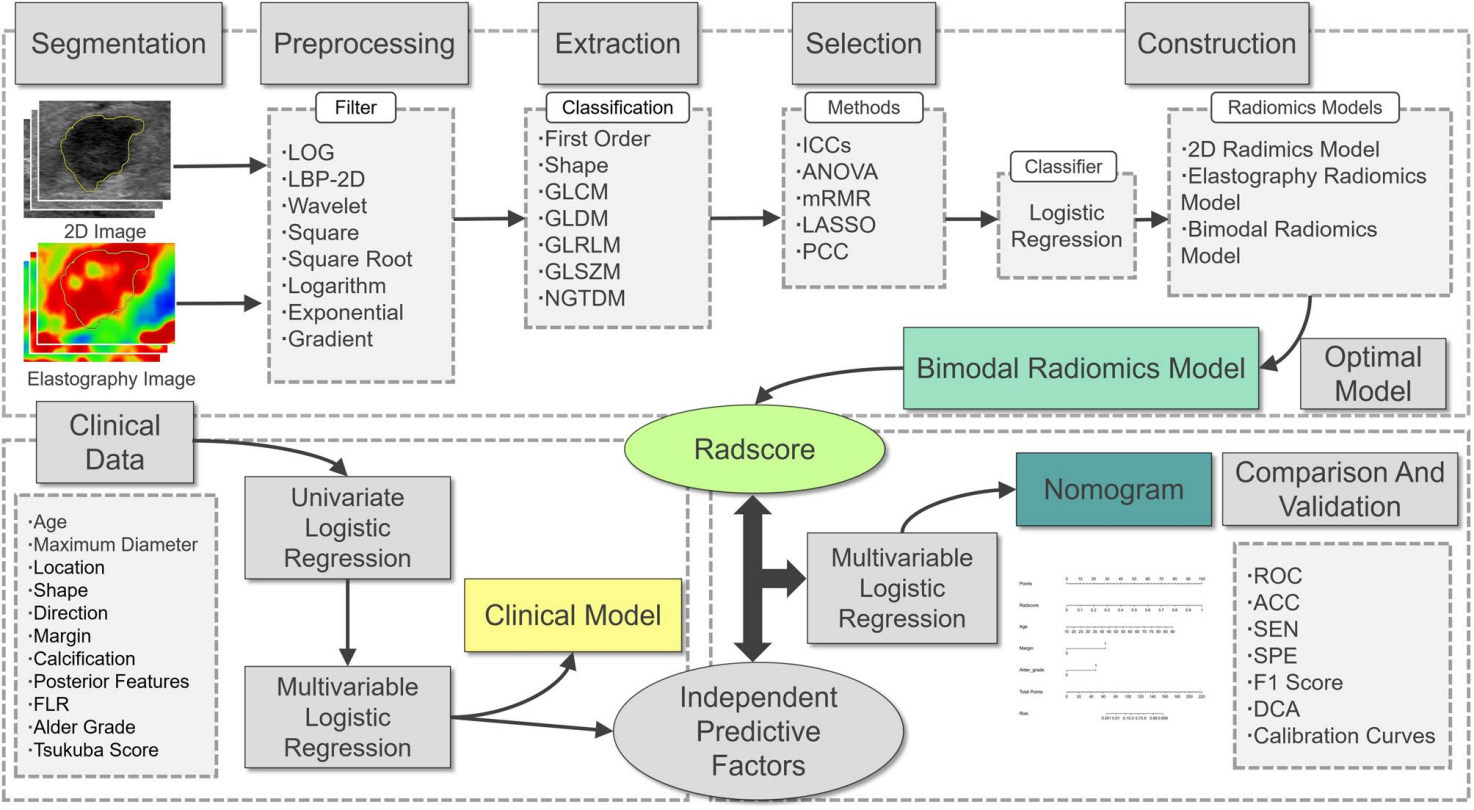
The workflow of this study.

With the booming development of radiomics, the application and research of radiomics on US images have become more and more extensive. Zhang et al. demonstrated that 2D US coupled with shear-wave elastography radiomics can improve the classification of BMs^10^. However, strain elastography based radiomics remains insufficiently studied in predicting BBMs and MBMs. The aim of this study was to construct and screen the best model for diagnosing the BBMs and MBMs based on the clinical and radiomics features of 2D and strain elastography US images. Figure 1 showed the flowchart of this study.

## Materials and methods

### Patients

This study was conducted in accordance with the Declaration of Helsinki and approved by the Medical Ethics Committee of the First Affiliated Hospital of Dalian Medical University (Approval No. PJ-KS-KY-2023-232). Due to the retrospective nature of the study, the need of informed consent was waived by the Medical Ethics Committee of the First Affiliated Hospital of Dalian Medical University. The data of patients who were diagnosed breast cancer between March 2021 and March 2023 were retrospectively collected for training and testing set. The inclusion criteria were as follows: (1) Sex was female. (2) The ACR BI-RADS grades of the BMs were 4 (including 4A, 4B, and 4C). (3) The BMs confirmed by puncture biopsy or surgical pathology. (4) Those who had complete 2D and strain elastography US images of the BMs.The exclusion criteria were as follows: (1) The patient was breastfeeding. (2) Those who had undergone or were undergoing radiotherapy or chemotherapy before the US examination. (3) The quality of images was extremely poor. In order to protect the patient’s privacy, information about the patient were desensitized before use.

### Clinical features and US images acquisition

Two sonographers with more than five years of experience in breast US diagnosis will independently review and record each patient’s age and maximum diameter, location, shape, direction, margin, calcification, posterior features, fat-to-lesions train ratio (FLR), Alder grade and Tsukuba score of the BMs without knowing the pathological result. Counting data that gave rise to different opinions were reviewed and decided by another expert with more than twenty years of work experience. The measurement data were finally averaged by the two sonographers.

The Philips EPIQ-7 US instrument with an L12-5 line array probe with a frequency range of 5–12 MHZ was utilized. 2D and strain elastography US images of patients were collected by a sonographer with more than 10 years of experience. The standard conventional breast US was followed by strain elastography. The color map of the strain elastography is set to red (no strain, strongest hardness), green (average strain, medium hardness), and blue areas (maximum strain, lowest hardness). The collection of the region of interest was set to include subcutaneous fat at the top and chest muscle at the bottom, with both sides more than 5 mm from the boundary of BMs.

### Images segmentation

Sonographer 1 and Sonographer 2 who had been trained to label all 2D and strain elasticity US images on the Darwin Research Platform without knowledge of the pathology results. One week later, both sonographers followed the same procedure for the second labeling. This study used intraclass correlation coefficients (ICCs) to evaluate intra- and inter-observer consistency, where ICCs less than 0.5 indicate poor reliability, between 0.5 and 0.75 indicate moderate reliability, and between 0.75 and 0.9 means good reliability, and greater than 0.90 means excellent reliability.

### Images preprocessing and feature extraction

A stratified sampling method was used to randomly divide 219 BMs into two independent sets in a ratio of 7:3, namely the training set (n = 153) and the testing set (n = 66). Before feature extraction, All images were resampled to 1 mm × 1 mm × 1 mm using the nearest neighbor interpolation algorithm to reduce spatial resolution inconsistencies caused by device differences or acquisition parameters, thereby improving feature comparability. Feature extraction in the Darwin research platform utilized the open source package Pyradiomics, which is used to extract radiomic features from medical imaging. Radiomic features can be divided into first-order, second-order and higher-order statistical features. On the Pyradiomics official website it is subdivided into seven groups: (1) first-order statistics; (2) based on shape (2D); (3) Gray Level Co-occurrence Matrix (GLCM); (4) Gray Level Dependence Matrix (GLDM); (5) Gray Level Size Zone Matrix (GLSZM); (6) Neighbouring Gray Tone Difference Matrix(NGTDM); (7) Gray Level Run Length Matrix(GLRLM). In order to extract more features, 8 filters (Wavelet, Square, Square Root, Laplacian of Gaussian (LOG), Logarithm, Exponential, local binary pattern (LBP) and gradient) performed filtering transformation on the original image, and then extracted first-order statistical features and grayscale texture features from the filtered image. The use of 8 filters enhances texture information across different scales or orientations (e.g., edges, speckles), improving the expressive capability of high-order features. Crucially, some filters (e.g., LOG, LBP, Wavelet) inherently provide denoising functions. For instance, the LOG filter enhances edge information by smoothing high-frequency noise, while the LBP operator suppresses homogeneous region noise while preserving structural features. In this study, there were 1125 radiomic features extracted from 2D and strain elastography US images respectively.

### Feature selection and Radscore

In order to make the algorithm converge faster, the original feature vectors were normalized to maximum and minimum values, that is, each dimension feature is linearly stretched to (0,1). Intraobserver and inter-observer consistency analysis was performed, and ICCs > 0.75 was used as the standard to remove the radiomics features with poor stability. Features with low linear correlation with labels were removed using ANOVA. Mutual information values and significance features were calculated for each feature using minimum redundancy and maximum relevance (mRMR). The mRMR was able to filters out features that have the highest correlation with the outcome but the lowest inter-correlation. Finally, the Least Absolute Shrinkage and Selection Operator (LASSO) was used to reduce redundant data and non-zero coefficients were applied to obtain stable features. To further optimize the hyperparameters of the prediction model and enhance its generalization ability, a hyperparameter tuning strategy integrating Grid Search with Cross-Validation was implemented. Specifically, for key hyperparameters such as l1_ratio and penalty factor C, predefined value ranges were exhaustively traversed via Grid Search. During training, tenfold cross-validation was employed to evaluate the performance of each parameter combination (e.g., accuracy, AUC). The optimal parameter set with the minimal cross-validation error was selected as the final model configuration. This approach effectively balances model complexity and overfitting risks, ensuring robustness on independent test datasets. The selected radiomics features and the weighted regression coefficients were linearly combined to obtain the radiomics scores (Radscore).

### Models construction

The clinical features were analyzed through univariate and multivariable logistic regression, independent predictive factors for identifying BBMs and MBMs were screened out, and a clinical model was established.

Using the screened imaging radiomics features, a model for predicting the BBMs and MBMs was constructed. Logistic regression classifiers were chosen to build the 2D radiomics model and elastography radiomics model respectively. The features of the two models were fused and the Pearson correlation coefficient (PCC) was calculated, and the features with multicollinearity, i.e., those with PCC > 0.80, were screened out to build a bimodal radiomics model and calculate the Radscore of the model. Multivariate logistic regression was analyzed for independent predictors and Radscore to construct joint predictive model nomogram.

### Statistical analysis

The prediction performance was assessed by AUC, accuracy (ACC), sensitivity (SEN), specificity (SPE),and F1 score. Differences between the AUCs of the different models were compared using the DeLong test. The agreement between the predicted and actual results of the five models was assessed using calibration curves, and the degree of fit of the five models was assessed using the Hosmer–Lemeshow test. To further assess the clinical application value of the five prediction models, Decision curve analysis (DCA) was used to quantify the standardized net benefit at different threshold probabilities. In order to verify the stability and generalisation of the nomogram, the ten-fold cross-validation method was applied to randomly divide the training set into ten groups, where every nine groups are the training set and the rest of the groups are the validation set, and the stability of the nomogram was assessed using the average AUC value of the ten results. All statistical analyses were performed with R software. The *t* test or Mann–Whitney *U* test was performed to compare continuous variables, while a *χ*^2^ or Fisher’s exact test was used for classifying variables between groups. Characteristics with intra-observer and inter-observer ICCs > 0.75 were screened by Python selection of two-way random effects model, single measure type and evaluation type of absolute consistency. Nomogram, DCA and calibration curves were sketched using the “rms” package, “rmda” package and “riskRegression” package in R software. The AUC for the five models were plotted using Medcalc software. All statistical tests were two sided, and *p* < 0.05 indicated significant.

## Results

### Patient characteristics

There were 153 and 66 BMs in the training and testing sets, respectively. In total, 133 and 86 patients with BBMs and MBMs, respectively. Patients’ clinical and pathological characteristics were summarized in Table 1 and Supplementary Table 1. Basic clinical features, including patients’ age and maximum diameter, location, shape, direction, margin, calcification, posterior features, fat-to-lesions train ratio (FLR), Alder grade and Tsukuba score of the BMs, were not statistically different between the training and testing sets (*p* > 0.05).

**Table 1:** Clinical characteristics of the training and testing sets.

Characteristics	Traingning set (N = 153)	Testing set (N = 66)	*p*
Age(years old)	49(41–59)	46(40–60)	0.526^a^
Maximum diameter(mm)	13(8–19)	12(8–18)	0.317^a^
Location			0.111^b^
Right	92	32	
Left	61	34	
Shape			0.526^b^
Regular	21	7	
Irregular	132	59	
Margin			0.782^b^
Circumscribed	85	38	
Not circumscribed	68	28	
Direction			0.619^b^
Parallel	140	59	
Not parallel	13	7	
Posterior features			0.263^b^
Not Shadowing	132	53	
Shadowing	21	13	
Calcification			0.427^b^
No	103	48	
Yes	50	18	
FLR	2.03(1.32–4.49)	2.23(1.56–4.74)	0.270^a^
Tsukuba score			0.890^b^
1–3	86	39	
4–5	67	27	
Alder grade			0.318^b^
0–I	103	42	
II–III	50	24	

Age was shown as mean ± standard deviation.

Abbreviations: FLR, fat-to-lesions train ratio.

^a^Student’s *t-*test or Mann–Whitney test.

^b^Chi square test or Fisher’s exact test.

### Establishment of clinical model

In order to screen out independent predictors, univariate logistic regression analysis was performed for clinical features. The results showed that there were statistically significant differences between the benign and malignant groups in age, maximum diameter, location, margin, posterior features, calcification, FLR, Tsukuba score and Alder grade (*p* < 0.05) (Table 2). These factors were included in multivariate logistic regression analysis and the clinical model was established. The results showed that age, maximum diameter, margin, FLR and Alder grade were independent predictors of MBMs.

**Table 2:** Results of univariate and multivariate logistic regression analysis based on clinical features.

Characteristics	Univariate logistic regression		Multivariable logistic regression	
	OR(95% CI)	*p*	OR(95% CI)	*p*
Age	1.106(1.074–1.140)	< 0.001	1.110(1.048–1.177)	< 0.001
Maximum diameter	1.157(1.103–1.214)	< 0.001	1.142(1.050–1.243)	0.002
Location	1.784(1.020–3.120)	0.042	0.833(0.224–3.103)	0.786
Shape	–	0.998		
Margin	42.940(19.047–96.803)	< 0.001	50.030(10.703–233.859)	< 0.001
Direction	2.534(0.990–6.484)	0.052		
Posterior features	3.466(1.612–7.455)	0.001	0.232(0.037–1.447)	0.118
Calcification	2.976(1.647–5.375)	< 0.001	1.620(0.407–6.441)	0.493
FLR	2.335(1.871–2.914)	< 0.001	1.949(1.229–3.093)	0.005
Tsukuba score	11.100(5.845–21.081)	< 0.001	2.506(0.463–13.553)	0.286
Alder grade	8.566(4.529–16.200)	< 0.001	7.831(1.957–31.334)	0.004

### Establishment of radiomics models and nomogram

1125 features were extracted from 2D ultrasonic images and strain elastography images, respectively, and the features with ICCs < 0.75 were screened out through ICCs calculation. The remaining features were selected by ANOVA, mRMR and LASSO feature selection methods, and finally three and eight radiomics features were selected to establish 2D radiomics models and elastography radiomics models, respectively. Then, the PCC between the six features extracted from the 2D images and the strain elastography US images is calculated. One feature with PCC > 0.80 is removed, and the remaining five features were fused to build a bimodal radiomics model and calculate its Radscore. Five features included in the bimodal radiomics model were: Sphericity, Interquartile Range, Run Length Non-Uniformity Normalized, Busyness, Gray Level Non-Uniformity.

The Radscore of the bimodal radiomics model was calculated as follows:

Radscore = −3.111 × Sphericity−2.924 × Interquartile Range + 2.026xBusyness−1.923 × Run Length Non-Uniformity Normalized + 1.347 × Gray Level Non-Uniformity + 2.142.

**Fig. 2:**
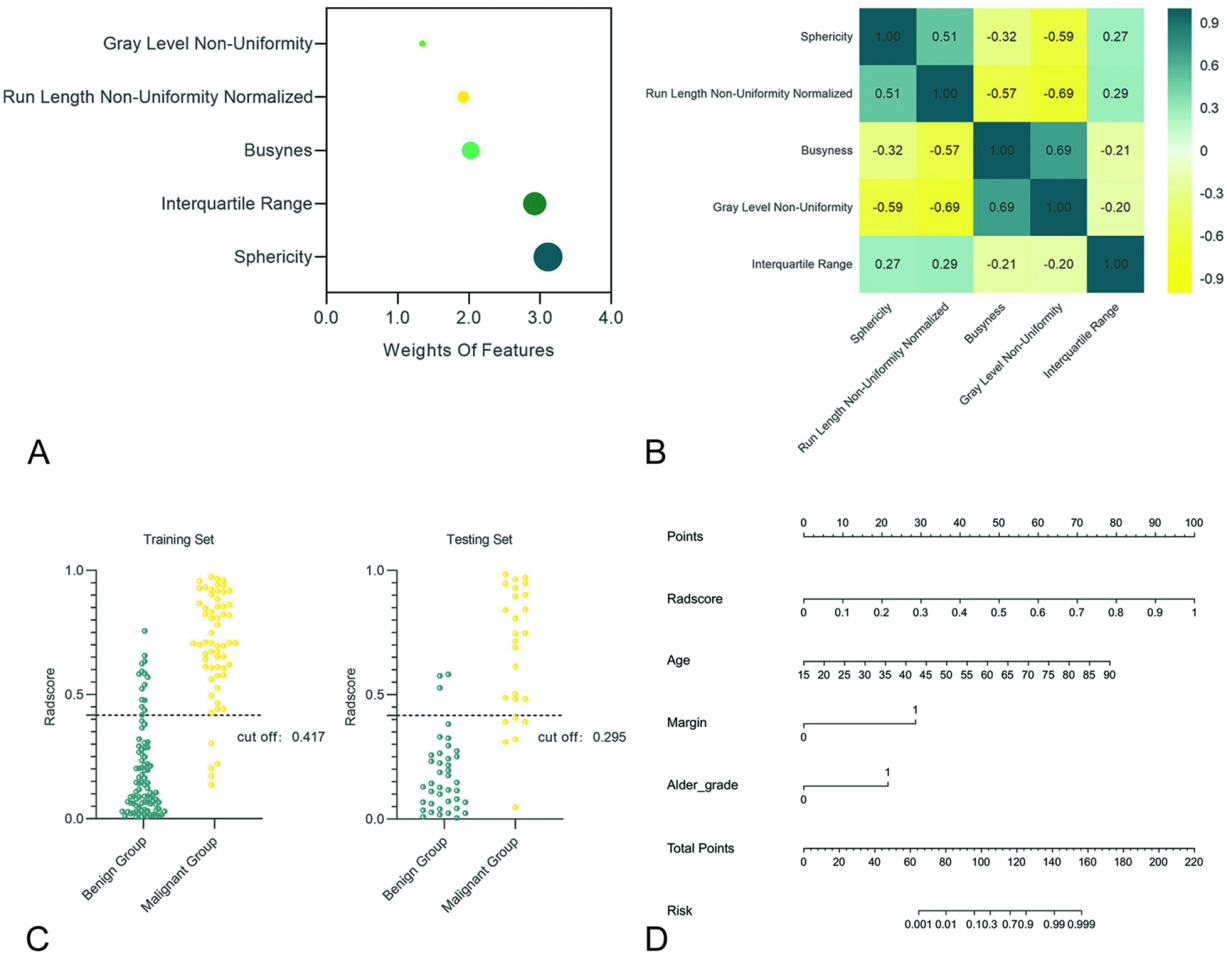
(**A**) Bubble diagram of the weights of the five features screened, from which it could be observed that Sphericity contributes the most to Radscore, followed by Interquartile Range. (**B**) Heatmap of the correlation between the five features, each value in the plot represented the PCC value between the corresponding two features. (**C**) Figure showed the distribution of Radscore in benign and malignant groups, when the cutoff value of Radscore in the training set and testing set was taken as 0.417 and 0.259, respectively, which could well distinguish BMs. (**D**) Nomogram constructed jointly by independent predictors and Radscore.

This five features and their weights for building Radscore were presented in Fig. 2A. Figure 2B shows a heat map of the correlation between these five features. Figure 2C illustrates the distribution of Radscore for the benign and malignant groups in the training and testing sets. Table 3 details the implications of these radiomics features and their correlation with MBMs. The distribution of the five features in the BBMs and MBMs were shown in Fig. 4.

**Table 3:** The significance of the five features selected in the bimodal radiomics model and their correlations.

Radiomics features	Category	Filter	From	Significance	Correlation
Sphericity	Shape2D	Original	2D images	A measure of the roundness of the shape of the tumor region relative to a circle	−
Busyness	NGTDM	Square Root	2D images	A measure of the change from a pixel to its neighbour	+
Run Length Non-Uniformity Normalized	GLRLM	LBP-2D	2D images	Measures the similarity of run lengths throughout the image	−
Gray Level Non-Uniformity	GLRLM	Wavelet-HL	Elastography images	Measures the similarity of gray-level intensity values in the image	+
Interquartile Range	First Order	Wavelet-LL	Elastography images	a method in descriptive statistics used to determine the difference between the third quartile and the first quartile	−

In order to improve the readability and clinical applicability of the prediction model, multivariate logistic regression was used to analyze the independent predictors of MBMs in clinical features and the Radscore of the bimodal radiomics model (Table 4), and a nomogram was constructed (Fig. 2D).

**Table 4:** Results of multivariate logistic regression analysis based on clinical features and Radscore.

Characteristics	Multivariable logistic regression	
	OR (95% CI)	P
Age	1.109(1.043–1.179)	0.001
Maximum diameter	1.049(0.928–1.186)	0.447
Margin	29.683(5.948–148.126)	< 0.001
FLR	1.553(0.955–2.526)	0.076
Alder grade	8.454(1.457–49.050)	0.017
Radscore	1042.992(17.749–61,290.285)	0.001

FLR, fat-to-lesions train ratio.

### Performance and comparison of models

**Fig. 3:**
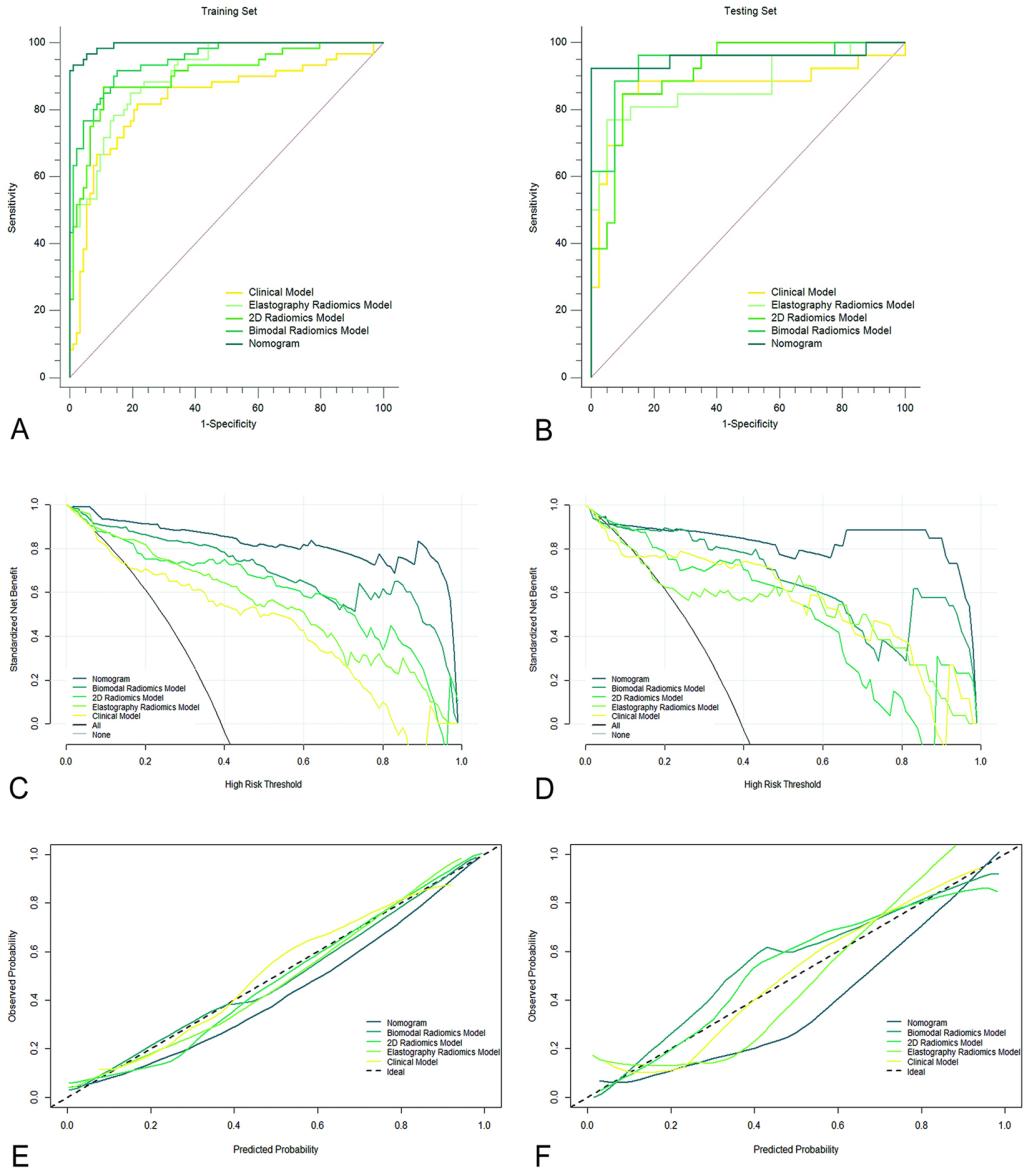
ROC curves, DCA curves and calibration curves for the five models in the training and test sets. (**A**) and (**B**) showed that the AUC values of nomogram in the training and testing sets were 0.99 and 0.95, respectively, outperforming the other four models. (**C**) and (**D**) indicated nomogram had gained more net benefit in predicting malignant breast masses. (**E**) and (**F**) showed that the gray line indicated perfect prediction under ideal conditions, and as the model curve got closer to the gray line, which indicated that the predictive ability of the model was getting better and better.

Figure 3A and B showed the prediction performance of the five models in the training and testing sets, respectively, and were summarized in Supplementary Table 2. In the training set, the AUC, accuracy, sensitivity, and F1 score of the nomogram were 0.98, 0.94, 0.97 and 0.93 respectively; In the testing set, the AUC, accuracy, sensitivity, and F1 score of the nomogram were 0.95, 0.95, 0.88 and 0.94 respectively. The performance of the nomogram was superior to that of other models. The results demonstrated that the AUC, accuracy, sensitivity and F1 score of the bimodal radiomics model in the training and testing sets were improved by different degrees compared with the 2D radiomics model and the elastography radiomics model, indicating that the prediction efficiency of the bimodal radiomics model was better than that of the two single-modal radiomics models. In addition, after combining the Radscore of the bimodal radiomics model with the clinical features to construct a nomogram, the predictors such as AUC, specificity, accuracy, and F1 score were improved to different degrees, which indicated that compared with clinical model, the ability to predict BMs and MBMs can be significantly improved by adding bimodal radiomics model. In the training set, the results of DeLong test illustrated that the AUC of the clinical model was significantly different compared with the AUC of the elastography radiomics model and the bimodal radiomics model, respectively (*p* < 0.05). There were significant differences in AUC between nomogram and clinical model, elastography radiomics model, 2D radiomics model and bimodal radiomics model (*p* < 0.05).

**Fig. 4:**
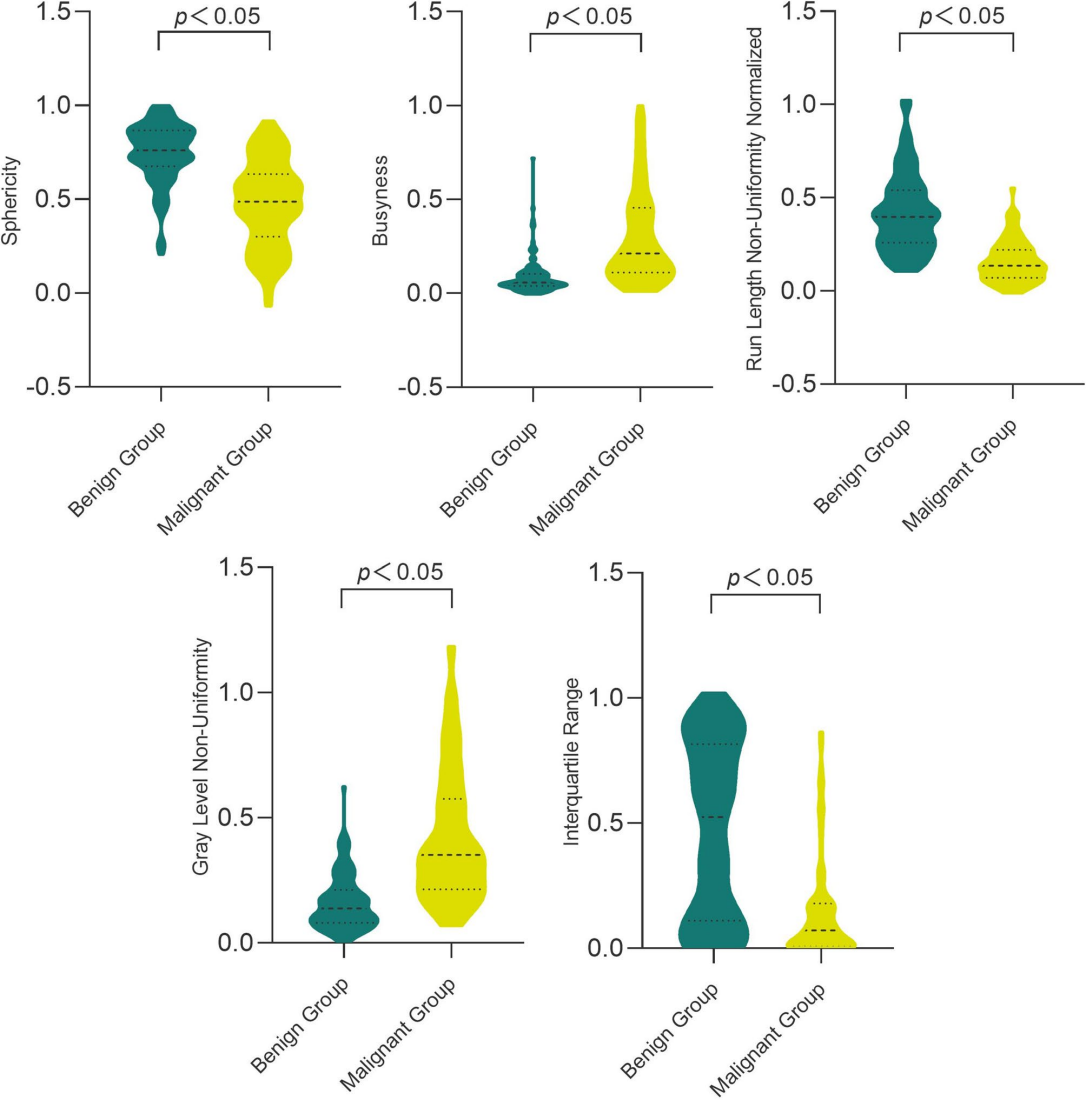
Distribution of the five characteristics in the benign and malignant groups of BMs, the middle dotted line indicated the median of the group, and the upper and lower dotted lines indicated the third quartile and the first quartile of the group, respectively.

The calibration curves of the five prediction models in the training and testing sets were shown in Fig. 3E and F, respectively. The gray line in the figure represents the perfect prediction efficiency under ideal conditions. The closer the model curve is to the gray line, the better the prediction ability of the model is. As can be observed from the figure, nomogram outperformed the other four models in both the training and testing sets. The calibration curves of the five models were verified using Hosmer–Lemeshow test, and the results revealed that except the testing set of the elastography radiomics model produced a significant (*p* < 0.05) result, the other models produced a non-significant (*p* > 0.05) result, providing good calibration evidence (Fig. 4).

**Fig. 5:**
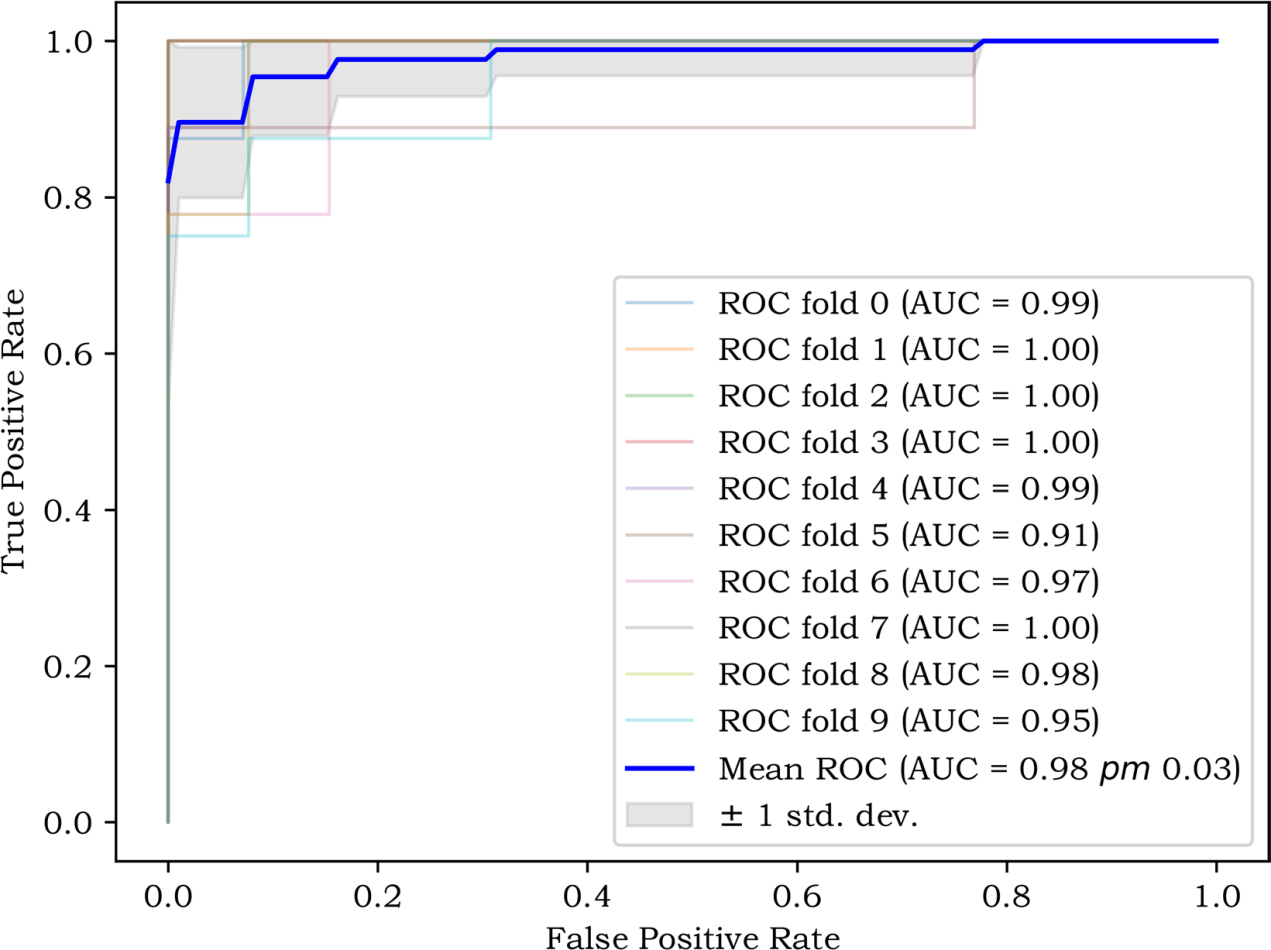
The mean value of ROC of Nomogram after ten-fold cross-validation was 0.98, which proved that the model had some stability in predicting the benign and malignant of BMs.

Figure 3C and D showed the DCA for the five prediction models in the training and testing sets, respectively, where the vertical axis represents the net benefit of standardization and the horizontal axis shows the probability of the corresponding risk threshold. In the testing set, nomogram can be used to predict BBMs and MBMs with a higher net benefit than the other four models when the threshold probability is between 0.10 and 1.00. The ROC mean value of the ten-fold cross-validation of the nomogram was 0.98 (Fig. 5), which confirmed that this model was stable.

## Discussion

This study discussed the use of radiomics based on 2D and strain elastography US images in the differential diagnosis of BBMs and MBMs. An easy-to-use graphic tool, the nomogram, was constructed in this study to facilitate the noninvasive assessment of BBMs and MBMs, thus supporting valuable information for clinical decision making.

BC screening can usually be performed by various medical imaging methods such as mammography, US and MRI^4^. However, the sensitivity and specificity of all current imaging tests in performing differential diagnosis of BMs is limited to some extent due to clinical and technical reasons. For example, in clinical practice, MRI has been suggested as the primary screening method for BC due to its non-ionising radiation damage, high soft tissue resolution, and advantages in identifying the location and size of lesions^11^. However, its specificity is limited by several factors affecting image quality, such as magnetic field and gradient strength^12^, coil performance^13^, contrast agent effect^14^ and menstrual cycle^15^. Although mammography is the mainstay of early BC detection, it has limited effectiveness in detecting suspicious lesions in dense breast tissue. The sensitivity and specificity of mammography have been reported to be approximately 90%^16^. As a complementary screening method for dense breast, US combined with mammography can identify up to 50% more BMs than mammography alone^17,18^.

US elastography was pioneered by Ophir et al. in 1991^19^. This elastography technique compensates for traditional B-mode US by superimposing stiffness measurements on spatial information. As a complementary modality to B-mode US, US elastography is easy to use and interpret, is more comprehensive in assessing stiffness differences between different tissue types, and can improve the accuracy of diagnosis of BMs^20^. Currently, US elastography improves the sensitivity of detection of small BMs^21^, shows a high degree of specificity for the diagnosis of BC and has become one of the preferred tests prior to breast invasive biopsy^22^. The two most commonly used elastography techniques are strain elastography and shear wave elastography. Several previous studies have reported that strain elastography and shear wave elastography may be able to improve the accuracy of identifying BBMs and MBMs and reduce unnecessary puncture biopsies^23,24^. The Tsukuba score has been widely used to differentiate BBMs and MBMs. For example, Itoh et al. showed that the sensitivity, specificity and accuracy of the Tsukuba score were 86.5%, 89.9% and 88.3%, respectively^25^. In a review of 22 strain elastography studies, most of which used the Tsukuba score, the overall mean sensitivity for the diagnosis of MBMs was 83% and the mean specificity was 84%^8^. The elastography strain ratio has been used to semi-quantitatively describe BMs as a more objective method of evaluation^26^. Strain ratios are calculated by dividing the mean strain in the normal reference tissue by the mean strain within the BMs. The choice of normal reference tissue is not clearly defined, and either normal breast glandular tissue or adipose tissue has been used as the reference tissue in several studies^27,28^. However, several studies have shown that FLR provides better diagnostic performance than glandular strain ratio^29,30^. This may be due to the fact that adipose tissue stiffness is essentially stable with little individual variation while breast glandular tissue stiffness may be influenced by a variety of factors such as sex hormones, menstrual cycle, and duration of breastfeeding.

Radiomics is a rapidly developing computer-aided technology that converts medical image information into a series of statistical data through computer algorithms^31^. Previous studies have shown that the microscopic features of images are closely related to the microscopic structure and biological behavior of tumors^32^. The imaging features reflect the texture of the tumor and are important biomarkers of tumor heterogeneity. As previously reported, the researchers established a Radscore containing 19 selected radiomics features to predict the malignancy of thyroid nodules. The AUC of the training group and the validation group in this study were 0.921 and 0.931, respectively, and the Radscore proved to have a good ability to distinguish malignant and benign thyroid nodules^33^. Radiomics holds significant promise in oncology research, with increasing attention being paid to the association between ultrasound radiomic features and tumor molecular subtypes, prognosis, and other relevant clinical outcomes. For instance, one study demonstrated the efficacy of ultrasound radiomic features in differentiating molecular subtypes of breast cancer and correlating with patient prognosis^34,35^. Another study revealed that ultrasound radiomic features can predict the response of breast cancer patients to neoadjuvant chemotherapy^36^. Furthermore, our published research has demonstrated that ultrasound radiomics can effectively predict Ki-67 expression levels in breast cancer, offering valuable insights for clinical decision-making^37^. These findings suggest that ultrasound radiomics has the potential to serve as a valuable tool in precision oncology.

In this study, a clinical model based on clinical features, a 2D radiomics model based on 2D US images, a elastography model based on strain elastography US images, and a bimodal radiomics model integrating two radiomics models were established to test the ability of the four models to distinguish BBMs and MBMs respectively. The results showed that the discrimination efficiency of the bimodal radiomics model was better than the other three models. Nomogram combined with multiple risk factors has been widely used to predict the prognosis and outcome of the disease. Huang et al. incorporated the imaging radiomics characteristics and clinical risk factors into the nomogram^38^. The nomogram is superior to clinical risk factors alone in predicting disease-free survival in early non-small cell lung cancer. In this study, Radscore of the bimodal radiomics model was independent predictors of MBMs along with patients’ age, FLR, and Alder grade. By combining the above variables, a visual tool called nomogram was constructed. The predictive performance of the nomogram for MBMs performed well in both the training and testing sets, with AUCs of 0.99 and 0.95, respectively, significantly outperforming the other four models. The specificity of the clinical model was lower than that of the elastography radiomics model and the 2D radiomics model. After the nomogram was established with Radscore, the specificity of the prediction model was significantly improved. The nomogram revealed that Radscore had a high contribution to this model, and age had also shown a significant contribution to the prediction efficiency of this model. The nomogram will eventually prove to be a good diagnostic model for clinicians to better distinguish BBMs and MBMs.

The Radscore in this study consisted of five radiomics features, including one first-order statistical feature, one shape feature and three texture features. From Fig. 2A, it can be seen that Sphericity contributes the most to Radscore, followed by Interquartile Range and Busyness. Therefore, Radscore may be intimately associated with the shape of BMs, and Radscore may be more reflective of the shape of BMs rather than the textural characteristics of BMs. The correlation heat map between the five features shows that the PCC between the five features is < 0.80 (Fig. 2B). Figure 2C reveals that when the cut-off of Radscore for the bimodal radiomics is 0.417 in the training set and 0.259 in the testing set, respectively, the differential diagnosis of the BBMs and MBMs was good. As shown in Fig. 4, the median Sphericity of the benign group was higher than that of the malignant group, reflecting that the BBMs were more circular in shape. The median Busyness in the malignant group was higher than that in the benign group, revealing that the brightness change rate between pixels in the malignant group and adjacent pixels was faster than that in the benign group.

## Conclusion

In conclusion, the 2D radiomics model and the elastography radiomics model were of diagnostic value in predicting BBMs and MBMs. The bimodal radiomics model based on 2D and strain elastography US images outperformed the single-modal radiomics models, demonstrating better predictive capability. Nomogram based on the bimodal radiomics model and clinical model could further enhance its effectiveness in the differential diagnosis of BBMs and MBMs, promoting non-invasive assessment of BBMs and MBMs, which might provide valuable information for clinical decision-making.

## Limitations of the study

Several study caveats should be acknowledged. First, the sample size of the study is relatively small, and it is expected that future studies with a larger sample size or by integrating open-access databases will be conducted to verify the results of this study. Secondly, the results obtain in this study have not been externally verified in other data sets, and multiple external data sets are needed for model validation in the future. Third, the study is a single-center retrospective study. Patient selection bias is inevitable and may affect the analysis results. While nomogram is developed in one hospital and have been well validated, more evidence is needed on the extent to which nomograms are applicable to patient populations in other hospitals, so more studies at multiple centers are needed in the future, and fourth, all images are taken from the same equipment. However, the study could not determine whether the models used were reproducible. A well-designed prospective cohort study will be necessary to confirm the effects of different US machines on model performance. Although the prediction of the nomogram could avoid unnecessary biopsies in patients with BBM, more research is needed to validate it.

## Supplementary Information

Below is the link to the electronic supplementary material.

Supplementary Material 1.

## Data Availability

The datasets generated or analyzed during the study are available from the corresponding author on reasonable request.

## Abbreviations

BMs: Breast masses
2D: Two-dimensional
ROC: Receiver operating characteristic
AUC: Area under the receiver operating characteristic curve
BC: Breast cancer
US: Ultrasound
BBMs: Benign breast masses
MBMs: Malignant breast masses
FLR: Fat-to-lesions train ratio
ICCs: Intraclass correlation coefficients
Radscore: Radiomics scores
PCC: Pearson correlation coefficient
ACC: Accuracy
SEN: Sensitivity
SPE: Specificity
DCA: Decision curve analysis
