# Supplementary material for: A study on the value of ultrasound strain elastography-based radiomics nomogram in the differential diagnosis of breast masses
Source: Sci Rep. 2025 Nov 27;15:45453. doi: 10.1038/s41598-025-29430-3 (PMC12749759; doi:10.1038/s41598-025-29430-3)
Supplement: Supplementary file 1 — Supplementary Material 1. [file 41598_2025_29430_MOESM1_ESM.docx]

***Supplementary Materials***

**Supplementary Tables**

## **Supplementary Table 1.** Pathology types of BMs included in the study

| Pathology Type | | Amount | % |
| --- | --- | --- | --- |
| BBMs |  | 136 |  |
|  | Adenosis | 27 | 19.9 |
|  | Hyperplastic disease of breast | 33 | 24.3 |
|  | Fibroadenoma | 64 | 47.0 |
|  | Mastitis | 3 | 0.2 |
|  | [Intraductal papilloma](http://www.baidu.com/link?url=yaXpp-e_Re_DnOlLEOXqREM-f4N3GJaUqphYMRe_z83ztIecP0m0rO_uRH9xGu1_NZtCe7tmFqZfTroef_Z_5keBeIojCy6C3skpz4ab2xC&wd=&eqid=e9534f2c000a7d0700000003676f9986) | 8 | 5.9 |
|  | Benign phyllodes tumors | 1 | 0.7 |
| MBMs |  | 83 |  |
|  | Intra-ductal carcinoma | 4 | 4.8 |
|  | Invasive ductal carcinoma | 78 | 94.0 |
|  | Lobular carcinoma in situ | 1 | 1.2 |

**Supplementary Table 2.** Comparison of the performance of five predictive models in the training and testing sets

| Models | Training set | | | | | |  | Testing set | | | | | |
| --- | --- | --- | --- | --- | --- | --- | --- | --- | --- | --- | --- | --- | --- |
|  | AUC(95%CI) | ACC | SPE | SEN | F1 score | Cut off |  | AUC(95%CI) | ACC | SPE | SEN | F1 score | Cut off |
| Clinical model | 0.82(0.74-0.89) | 0.79 | 0.81 | 0.76 | 0.74 | 0.43 |  | 0.86(0.75-0.97) | 0.86 | 0.88 | 0.85 | 0.83 | 0.49 |
| Elastography radiomics model | 0.91(0.85-0.95) | 0.84 | 0.86 | 0.80 | 0.79 | 0.54 |  | 0.88(0.77-0.94) | 0.87 | 0.95 | 0.76 | 0.83 | 0.54 |
| 2D radiomics model | 0.92(0.87-0.96) | 0.88 | 0.88 | 0.86 | 0.84 | 0.50 |  | 0.90(0.79-0.95) | 0.86 | 0.87 | 0.84 | 0.83 | 0.48 |
| Bimodal radiomics model | 0.96(0.92-0.99) | 0.89 | 0.86 | 0.93 | 0.87 | 0.51 |  | 0.93(0.85-0.98) | 0.89 | 0.85 | 0.96 | 0.87 | 0.35 |
| Nomogram | 0.98(0.96-1.00) | 0.94 | 0.93 | 0.97 | 0.93 | 0.50 |  | 0.95(0.88-1.00) | 0.95 | 1 | 0.88 | 0.94 | 0.53 |
